# Different contributions of plant diversity and soil properties to the community stability in the arid desert ecosystem

**DOI:** 10.3389/fpls.2022.969852

**Published:** 2022-08-25

**Authors:** La-Mei Jiang, Kunduz Sattar, Guang-Hui Lü, Dong Hu, Jie Zhang, Xiao-Dong Yang

**Affiliations:** ^1^College of Ecology and Environment, Xinjiang University, Ürümqi, China; ^2^Key Laboratory of Oasis Ecology of Education Ministry, Xinjiang University, Ürümqi, China; ^3^Xinjiang Jinghe Observation and Research Station of Temperate Desert Ecosystem, Ministry of Education, Jinghe, China; ^4^Xinjiang Uygur Autonomous Region Forestry Planning Institute, Ürümqi, China; ^5^College of Life Science, Northwest University, Xi’an, China; ^6^College of Geography and Tourism Culture, Ningbo University, Ningbo, China

**Keywords:** alpha diversity, beta diversity, diversity-stability relationship, stabilization mechanisms, spatial scale

## Abstract

As a one of the focuses of ecological research, understanding the regulation of plant diversity on community stability is helpful to reveal the adaption of plant to environmental changes. However, the relationship between plant diversity and community stability is still controversial due to the scale effect of its influencing factors. In this study, we compared the changes in community stability and different plant diversity (i.e., species, functional, and phylogenetic diversities) between three communities (i.e., riparian forest, ecotone community, and desert shrubs), and across three spatial scales (i.e., 100, 400, and 2500 m^2^), and then quantified the contribution of soil properties and plant diversity to community stability by using structural equation model (SEM) in the Ebinur Lake Basin Nature Reserve of the Xinjiang Uygur Autonomous Region in the NW China. The results showed that: (1) community stability differed among three communities (ecotone community > desert shrubs > riparian forest). The stability of three communities all decreased with the increase of spatial scale (2) species diversity, phylogenetic richness and the mean pairwise phylogenetic distance were higher in ecotone community than that in desert shrubs and riparian forest, while the mean nearest taxa distance showed as riparian forest > ecotone community > desert shrubs. (3) Soil ammonium nitrogen and total phosphorus had the significant direct negative and positive effects on the community stability, respectively. Soil ammonium nitrogen and total phosphorus also indirectly affected community stability by adjusting plant diversity. The interaction among species, functional and phylogenetic diversities also regulated the variation of community stability across the spatial scales. Our results suggested that the effect of plant diversities on community stability were greater than that of soil factors. The asynchronous effect caused by the changes in species composition and functional traits among communities had a positive impact on the stability. Our study provided a theoretical support for the conservation and management of biodiversity and community functions in desert areas.

## Introduction

Stability is a comprehensive feature of plant community structure and function (Wang, 2002), which is directly related to biodiversity maintenance, water and carbon balance, climate regulation, and soil and water conservation (Li et al., 2022). Under the influence of climate change and biological invasion, community stability was decreasing globally (Wang C. et al., 2021). Assessing its change and the influencing factors has become one of the most important topics in the scientific community (Li et al., 2022).

Plant diversity is considered as a key indicator to measure community stability, because ecosystem function and structure are increasingly vulnerable to the threat of biodiversity loss (Evans et al., 2022). However, the vast majority of previous studies used species diversity to assess community stability (Sasaki and Lauenroth, 2011; Craven et al., 2018; Liu et al., 2019). Since species diversity ignores the differences in functional traits and phylogenetic classification among species, it may have some shortages in assessing community stability (Chao et al., 2014). With the deepening of the understanding of biodiversity, an increasing number of ecologists have realized that functional and phylogenetic diversity are more advantageous than species diversity in assessing community stability (Su et al., 2021; Token et al., 2022). In addition, although studies have demonstrated that diversity can affect community stability in many ways, the influencing mechanisms and their relative contribution remain unclear (Sasaki and Lauenroth, 2011; Hallett et al., 2014). To date, scientists have identified at least four potential mechanisms of diversity for maintaining community stability: portfolio effect (higher diversity or richness leads to higher stability) (Hector et al., 2010); Selection effect (higher advantages lead to higher stability) (Grman et al., 2010); Insurance effect (asynchronous or compensatory dynamics of larger species lead to higher stability) (Brown et al., 2016); and over-yielding effect (larger total biomass leads to higher stability) (Hector et al., 2010). However, these mechanisms are not fixed, which varies among ecosystems and their contributions varies with the environment. The relationship between plant diversity and community stability needs to be further explored in diverse ecosystems.

Biodiversity can be divided into α and β diversity based on the regional scale (Jost, 2007; Ricotta and Szeidl, 2009). α diversity represents the number and distribution uniformity of species, and the variation on their functional traits in a community (Rosauer et al., 2009; Mason and de Bello, 2013). β diversity refers to the differences in species composition, evolutionary relationship and functional attributes among communities across spatial or temporal scales (Tan et al., 2019; Wang S. et al., 2021). In terms of the relationship between plant diversity and community stability, most studies focus on α diversity while ignore β diversity because the former is easy to observe, and the influences of spatial and temporal changes are not considered (Wang and Loreau, 2016; Qiao et al., 2022). Although some recent studies have tested the effect of β diversity on community stability at multiple environmental gradients (Zhang et al., 2019; Hautier et al., 2020; Wang S. et al., 2021), they have been concentrated in resource-rich environments, such as tropical and subtropical forests (Hautier et al., 2020; Wang S. et al., 2021; Qiao et al., 2022). Most of them did not analyze the effect of diversity on community stability across different spatial scales.

Community ecologists have long been interested in unraveling the maintenance mechanism of community stability in highly variable environments (Loreau and de Mazancourt, 2013; Li and Stevens, 2017; Li et al., 2018a). The possible influence of abiotic factors on community stability may be increased seriously due to the increasing global environmental change (De Laender et al., 2016; Wu et al., 2021). Recent studies have shown that abiotic factors (i.e., soil properties) may obscure the extent and direction of biodiversity’s impacts on community function and stability (Coelho de Souza et al., 2019; Wang et al., 2020). Environmental changes not only directly affect the community stability, but also indirectly affect it through changes in plant diversity (Xu et al., 2015b). For example, environmental change can greatly alter the composition, abundance and diversity of communities, thus affecting the maintenance of community stability (Hallett et al., 2014; Donohue et al., 2016). However, most of these studies have focused on forests and grassland ecosystems in moist regions, while few studies have been carried out in arid deserts (Hautier et al., 2020; Wang C. et al., 2021; Qiao et al., 2022). It is well known that one of the most striking features of desert ecosystems, that distinguishes them from other systems, is that local plants are subjected to severe drought stress due to the lack of rainfall (Gong et al., 2019; Song et al., 2020; Yang X. et al., 2022). In this case, the relationship between diversity and community stability in arid desert may be different from other systems.

The Ebinur Lake Wetland National Nature Reserve (ELWNNR) is a treasure house of biodiversity in arid desert areas of the Xinjiang Uygur Autonomous Region in the NW China, which provides a good place for studying the maintenance of diversity on community stability in arid desert ecosystem (Jiang L. et al., 2022). Affected by arid climatic conditions, the local ecosystem is relatively sensitive and vulnerable to environmental changes (Yang et al., 2019; Token et al., 2022). Although the relationship between plant diversity and community stability in arid desert system have been reported, they mainly focus on the effects of species diversity and functional diversity on community stability under different water and salt gradients, whereas did not discuss the influences of phylogenetic and beta diversity. Also, they did not consider the variation on the relationship between diversity and community stability along different spatial scales (Wang et al., 2017; Hu et al., 2021; Token et al., 2022). In order to solve these problems, we investigated community characteristics and diversity (α and β diversities) on the River bank, the transition zone and the desert hinterland in the ELWNNR, respectively. Then, based on different spatial scales (10 m × 10 m, 20 m × 20 m, and 50 m × 50 m), we compared the differences in plant diversity and community stability among three sampling sites, and the variation across three scales. After that, the structural equation model (SEM) is used to analyze the direct and indirect effects of plant diversity and soil properties on the community stability. Our study is aim to: (1) reveal the variation on the relationship between plant diversity and community stability across spatial scales; and (2) expose the regulation mechanism of soil properties and plant diversity on community stability in arid desert ecosystem. Our study can not only enrich people’s knowledge of the influencing factors and maintenance mechanisms of community stability, but also have important practical significance for the restoration and reconstruction of degraded arid desert ecosystems.

## Materials and methods

### Study area

**FIGURE 1:**
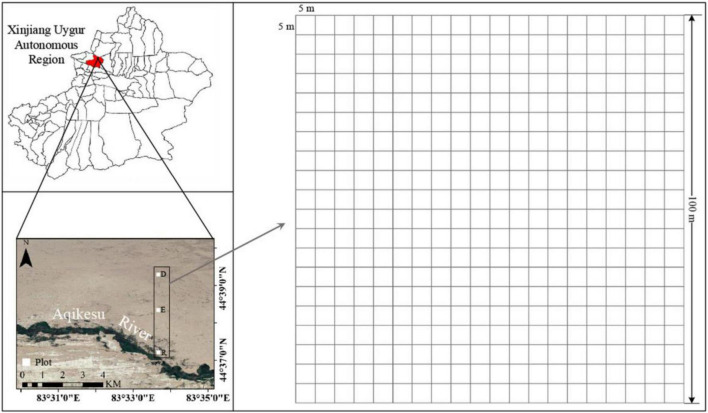
Study area and location of the sampling plots. R, E, and D represent riparian forest, ecotone community, and desert shrubs, which grow in the river bank, the transitional zone and desert hinterland, respectively.

The ELWNNR is located in the southwest of the Junggar Basin in the Xinjiang Uygur Autonomous Region, NW China (Figure 1). It is the lowest depression and water-salt accumulation center in the southwest margin of the Junggar Basin. Affected by a typical temperate continental arid climate, the annual evaporation is more than 1600 mm, the annual rainfall is about 100 mm, and the sunshine duration is about 2800 h. The extreme maximum and minimum temperatures are 44 and -33^°^C, respectively (Hu et al., 2022). The local community is mainly consisted of desert plants (Yang et al., 2014). The main dominant plants include *Populus euphratica*, *Haloxylon ammodendron*, *Halimodendron halodendron*, *Reaumuria soongarica*, *Nitraria roborowskii*, *Halocnemum strobilaceum*, *Alhagi sparsifolia*, *Apocynum venetum*, *Seriphidium terrae-albae*, *Phragmites australis*, *Salsola collina*, *Suaeda glauca*, etc. (Jiang L. et al., 2022). Three large sample plots of 1 hm^2^ (100 m × 100 m) was set up in the river bank, the transition zone between riparian forest and desert shrubs (ecotone community), and the desert hinterland in the ELWNNR, respectively. Each sample plot was divided into 400 of 5 m × 5 m quadrats (Figure 1). *P. euphratica* was the dominant species in the riparian forest, *H. ammodendron* and *P. euphratica* were the co-dominant species in ecotone community, while *H. ammodendron* was the dominant species in the desert shrubs. The main species of three communities were shown in the appendix (Supplementary Tables 1–3).

### Community investigation and leaf functional trait determination

Plant community characteristics (species composition, abundance, plant height, and crown area) were investigated in all quadrats. Then, 20 mature leaves were collected randomly from each individual in each quadrat. The leaf length, width and thickness were measured by vernier caliper. The leaf area was calculated by grid method. More specifically, the leaves were spread on a 1 mm^2^ grid paper and photographed with a camera. The images were then uploaded to Photoshop 7.0 to calculate the leaf area. After all leaves were brought back to the laboratory, an electronic analytical balance with accuracy < 0.0001 g was used to measure leaf fresh weight. The drying method was used to measure leaf dry weight and then calculated the specific leaf area and dry matter content. After all dry leaves were ground and sieved into powder. The contents of leaf carbon, nitrogen and phosphorus were determined using the potassium dichromate dilution heat method, the Mo-Sb colorimetric method and the Kjeldahl digestion method.

### Collection and measurement of soil samples

In each quadrat, the diagonal method was used to select the center position. After considering that 0–20 cm topsoil can well reflect soil nutrient status in arid desert (Tian et al., 2010), we drilled this topsoil in the center position in this study. Two soil samples were collected from each quadrat, one of which was collected with a ring knife to measure its fresh weight and soil bulk density, and then brought back to the laboratory for drying to calculate soil water content (SWC). The other sample was brought back to the laboratory to dry naturally for later soil properties determination. SWC was determined by the drying method (Zhang et al., 2015). Soil salinity content (SA) and soil pH was determined using the weight method and a pH meter (Leici PHS-3C, Shanghai Yidian Scientific Instrument Co., Ltd, China), respectively. Soil organic carbon (SOC), total nitrogen (TN), ammonium nitrogen (AN), and nitrate nitrogen (NN) was determined by the potassium dichromate dilution heat method, the Kjeldahl digestion method (BUCHI-K370, BUCHI Labortechnik AG, Switzerland) (Dalai et al., 1984), the indophenol blue colorimetry (Bao, 2000), and the dual-wavelength ultraviolet spectrophotometry (Norman et al., 1985). The total phosphorus (TP) and available phosphorus (AP) was determined by the Mo-Sb colorimetric method (Spectrophotometer UV1200, Shanghai AOE Instruments Co., Ltd., China) (Li et al., 2019a).

### Statistical analysis

#### Spatial scales

In order to test whether the relationship between community stability and diversity varied with spatial scales (scale-dependence), 5 m × 5 m quadrat was used as the basic unit and then randomly generated plots with area of 10 m × 10 m, 20 m × 20 m, and 50 m × 50 m, respectively. The number of each new plot types was 100. The values of soil properties and plant functional traits of each newly generated plot were expressed by means of the average of 5 m × 5 m basic quadrats (Hu et al., 2022). All the above processes were completed in R4.1.2 software.

#### Community stability

Plant community stability was determined by using inverse of coefficient of variation (ICV) (Eq. 1). Where μ is the average density of each species in the quadrat; σ is the standard deviation of each species density. Larger ICV value suggest higher community stability and small variability of species density (Wu et al., 2014; Yang et al., 2017).


(1) $$\text{ICV}=\frac{\mu }{\sigma }$$

#### Plant diversity

Species and functional diversities were completed in *Vegan* and *FD* package. The phylogenetic trees were firstly generated using the maximum likelihood (ML) method and the Bayesian inference (BI) method implemented MEGA 6.0 and BEAST 1.7, respectively (Drummond et al., 2012). The results of the phylogenetic trees of the three communities were presented in the appendix (Supplementary Figures 1–3). Then, the *picante* software package was used to calculate phylogenetic diversity. As suggested from Tan et al. (2019), species β diversity was divided into the Local Contribution to Beta Diversity (LCBD) and the Species Contribution to Beta Diversity (SCBD). Species β diversity, LCBD and SCBD were completed in *adespatial* package. All calculations of diversity were conducted in R 4.1.2.

#### Influencing factors of diversity-stability relationship

Differences in community stability and diversity among three communities (riparian forest, ecotone community, and desert shrubs), as well as among three spatial scales were analyzed by the one-way ANOVA. The correlations among diversity, soil properties and community stability were analyzed by cor.test function. The relative contributions of soil properties (soil water content, salt content, organic carbon, pH and total nitrogen, etc.) and plant diversity (species diversity, functional diversity, phylogenetic diversity index, etc.) on community stability were analyzed using SEM. Before the calculation of SEM, the multiple linear regression model (MLRM) was used to reduce the number of variables while screen the influencing factors of community stability. The factors with VIF > 10 will be eliminated. However, since there were still many factors left over after the selection of the multiple linear regression model, the Principal Component Analysis (PCA) was continued to screen out the final influencing factors. One-way ANOVA, cor.test, MLRM and PCA were conducted using R 4.1.2, whereas SEM was completed in Amos 24.0.

## Results

### Change in community stability across spatial scales

**FIGURE 2:**
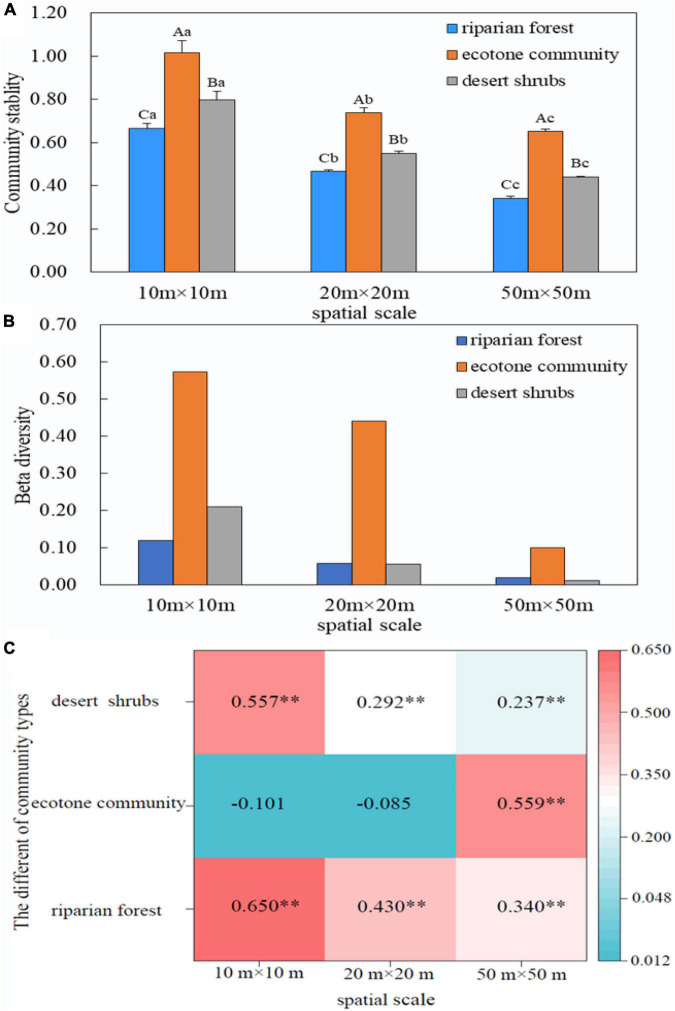
Relationship between community stability and β diversity. **(A)** Variation of community stability across spatial scales; **(B)** Variation of β diversity across spatial scales; **(C)** Relationship between LCBD and community stability. The capital letters indicate the difference of the stability between different communities at the same spatial scale, while the lower letters suggest the difference of the stability of the same community among different spatial scales. *P* < 0.05. ^**^P < 0.01.

Our results found that community stability decreased significantly with the spatial scales (100 m^2^ > 400 m^2^ > 2500 m^2^) in all sampling positions (*P* < 0.05). The order of community stability among three communities was as follows: ecotone community > desert shrubs > riparian forest (Figure 2).

### Change in plant diversity across spatial scales

**FIGURE 3:**
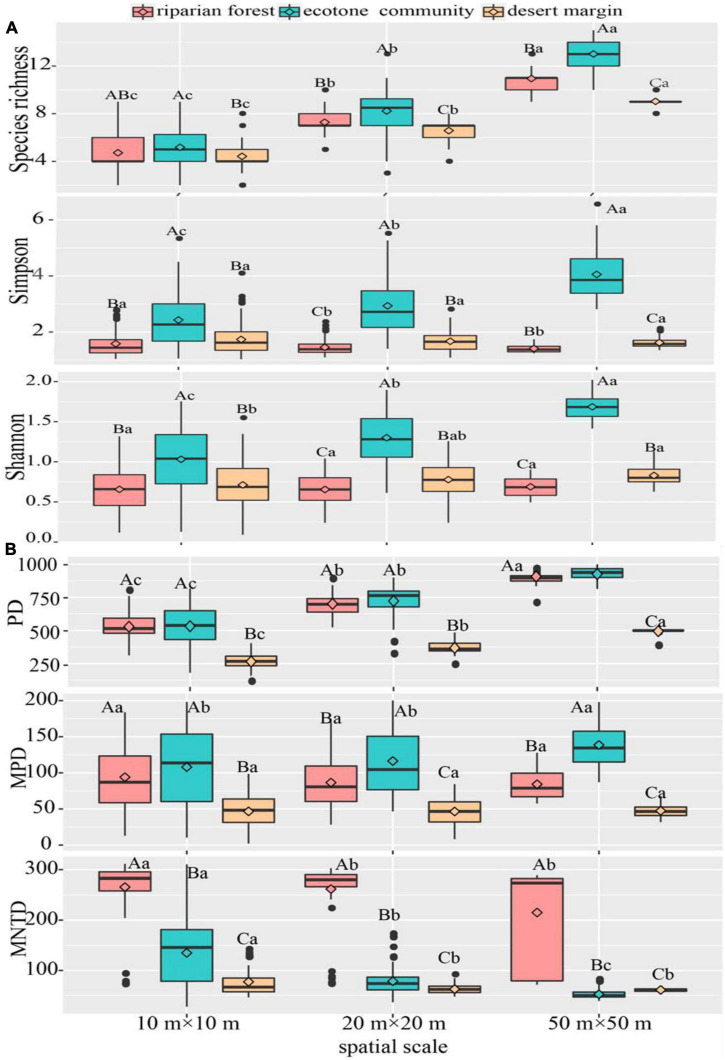
Variation of species and phylogenetic diversities across spatial scales. **(A)** Species diversity; **(B)** phylogenetic diversity. The instruction of capital and lower-case letters see Figure 2. *P* < 0.05.

Species richness and Shannon Wiener index increased with spatial scales in all sampling communities. The Simpson index showed the different pattern among three communities, which increased with the spatial scales in the ecotone community, but showed the opposite results in desert shrubs and riparian forest. Among three sampling positions, all species diversity (i.e., richness, Shannon Wiener, and Simpson indexes) were higher in the ecotone community than desert shrubs and riparian forest across three spatial scales (Figure 3).

With regarding to phylogenetic diversity, the phylogenetic richness (PD) showed an increasing trend with spatial scale in all sampling positions, while the mean nearest taxa distance (MNTD) decreased. However, the MPD increased with spatial scale in the ecotone community, while not changed in the riparian forest and desert shrubs. Among three sampling positions, PD and MPD were higher in the ecotone community and riparian forest than desert shrubs across three spatial scales, whereas MNTD showed the opposite pattern (Figure 3).

**FIGURE 4:**
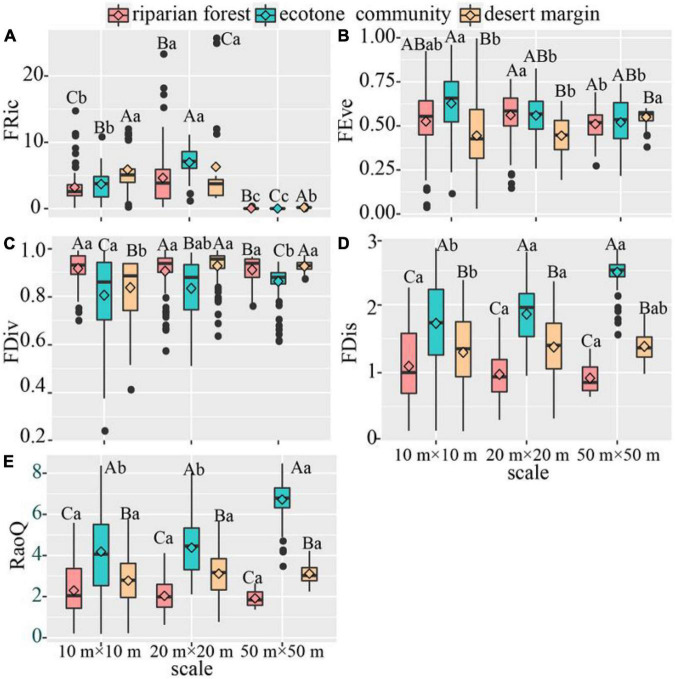
Variation of functional diversity across spatial scales. The instruction of capital and lower-case letters see Figure 2. **(A)** FRic. **(B)** FEve. **(C)** FDiv. **(D)** FDis. **(E)** RaoQ. *P* < 0.05.

In terms of functional diversity, our results showed that functional richness (FRic) of three sampling positions firstly increased and then decreased with the increase of spatial scales. The functional evenness (FEve) of riparian forest and ecotone community decreased with the increase of spatial scale, whereas desert shrubs showed an opposite pattern. Functional divergence (FDiv) and functional dispersion (FDis) increased with spatial scale in the ecotone community and desert shrubs, while not changed in the riparian forest. The RaoQ increased in the ecotone community, while not changed in the riparian forest and desert shrubs. Among three sampling positions, RaoQ, FDis and FEve were higher in the ecotone community than desert shrubs and riparian forest across three spatial scales, whereas FDiv showed the opposite pattern. FRic at 20 m × 20 m in the ecotone community was higher than that in desert shrubs and riparian forest, while showed the opposite pattern at 10 m × 10 m and 50 m × 50 m (Figure 4).

The β diversity decreased with the increase of spatial scales in all sampling positions. The β diversity in the ecotone community was larger than that in riparian forest and desert shrubs (Figure 2).

### Correlation between plant diversity and community stability

**FIGURE 5:**
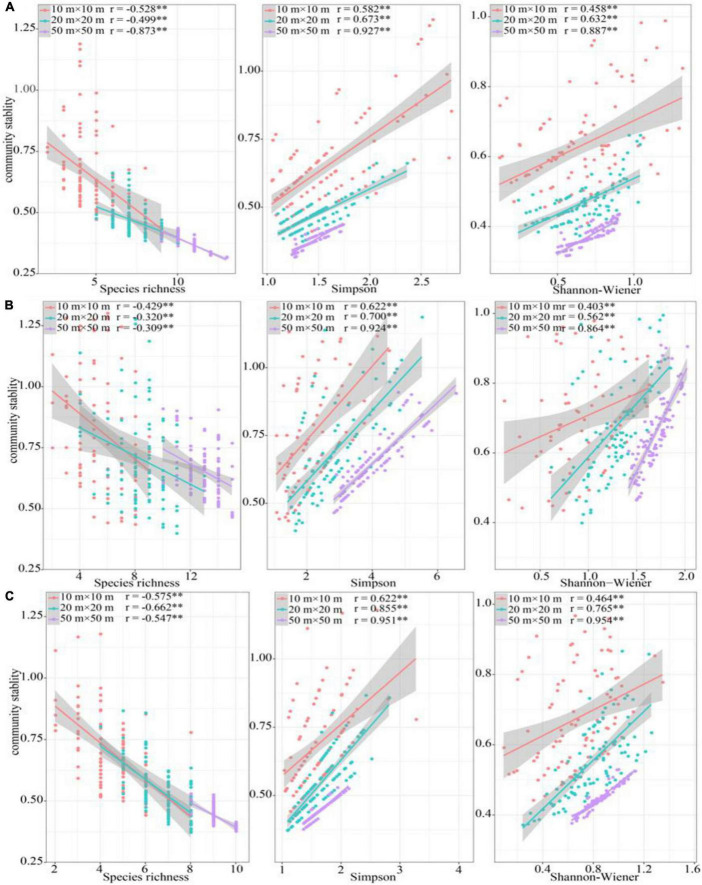
Relationship between species diversity and community stability. **(A)** riparian forest, **(B)** ecotone community, **(C)** desert shrubs. *P < 0.05, **P < 0.01, ***P < 0.001.

Richness has a negative correlation with community stability in all sampling positions across three spatial scales, while the Simpson and Shannon-Wiener indices were positively correlated (Figure 5). In most cases, the correlation coefficient between species diversity and community stability increased with increasing spatial scale (Figure 5).

Expect 50 m × 50 m in riparian forest, FRic had the significant negative relationship with community stability in the other sampling positions across three spatial scales. The relation of FDiv, FDis, and RaoQ to community stability was completely opposite. FDiv negatively correlated with community stability in all sampling positions across three spatial scales, but FDis and RaoQ were positive correlated (Table 1). The relationship of FEve with community stability showed no significant trend at different sampling positions and different scales. For example, it was significantly correlated with community stability in the ecotone community at 50 m × 50 m. However, in at the same size, it was significantly positively correlated with stability in the riparian forest. Unlike species diversity, the correlation coefficients between functional diversity and community stability had not showed a consistent change trend with the increase of spatial scale (Table 1).

**TABLE 1:** Relationship between functional diversity and community stability.

Types	Scale	FRic	FEve	FDiv	FDis	RaoQ
Riparian forest	10 m × 10 m	−0.421**	−0.142	−0.527**	0.597**	0.538**
	20 m × 20 m	−0.420**	−0.084	−0.254**	0.346**	0.304**
	50 m × 50 m	0.306**	0.332**	−0.126	0.278**	0.246**
Ecotone community	10 m × 10 m	−0.336**	0.313**	−0.475**	0.148	0.205*
	20 m × 20 m	−0.175	0.071	−0.394**	0.357**	0.266**
	50 m × 50 m	−0.412**	−0.507**	−0.658**	−0.050	−0.139
Desert shrubs	10 m × 10 m	−0.456**	0.194	−0.364**	0.568**	0.485**
	20 m × 20 m	−0.510**	−0.138	−0.535**	0.885**	0.853**
	50 m × 50 m	−0.547**	−0.068	−0.248*	0.933**	0.908**

*P < 0.05, **P < 0.01, ***P < 0.001.

**FIGURE 6:**
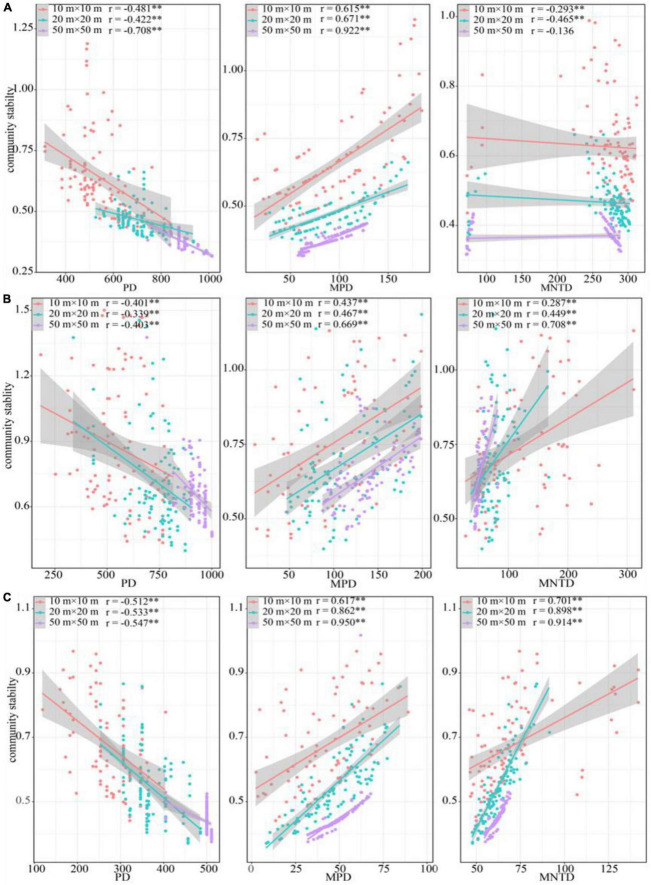
Relationship between phylogenetic diversity and community stability. **(A)** Riparian forest. **(B)** Ecotone community. **(C)** Desert shrubs. **P < 0.01.

In terms of phylogenetic diversity, PD was significant negative correlation with community stability in all sampling positions across three spatial scales, while MPD was positive related. MNTD was negatively correlated with community stability in the ecotone community and the desert shrubs, and positively correlated with community stability in the riparian forest (Figure 6). Similar to species diversity, the correlation coefficient between phylogenetic diversity and community stability increased with increasing spatial scale in most cases (Figure 6).

There was a significant positive correlation between LCBD and community stability in riparian forest and desert shrubs. The correlation coefficient between LCBD and stability in these two positions decreased with the increase of spatial scale. In the ecotone community, the relationship between beta diversity and stability varied greatly with scale, which showed no significant negative correlation at small and medium scales (10 m × 10 m and 20 m × 20 m), but significant positive correlation at 50 m × 50 m (Figure 2).

As shown above, beta diversity included LCBD and SCBD. Where SCBD represented the contribution of species to beta diversity. Within each scale, there was only one value of SCBD for each species. Thus there were only 3 values at 3 spatial scales, resulting in that the correlation analysis could not be used to judge the relationship between SCBD and community stability. In this study, however, we classified species into woody plants and herbaceous using life forms. Then, the changes of SCBD of these two types with spatial scale were used to determine whether they had an impact on community stability. Our results showed that the uniqueness of herbaceous and woody plants across spatial scales showed an opposite change in all sampling positions (Supplementary Figure 4). This indicated that SCBD can also affect community stability in arid and desert areas.

### The relative effects of plant diversity and soil factors on the stability of desert plant communities

The collinearity results of the MLRM showed that the following factors (species richness, PD, FRic, FEve, FDiv, MPD, MNTD, LCBD, total phosphorus and ammonium nitrogen) were filtered to be the influencing variances of diversity-community stability relationship (VIF<10) (Supplementary Table 4). After that, in order to further reduce the number of variables about plant diversity, the PCA was used to compress the latitude of variables. The results showed that the diversity was reduced to three principal components. PC1 was mainly composed of FRic, PD and species richness (Plant PC1). PC2 included FDiv, MPD, and LCBD (Plant PC2), while PC3 included MNTD and FEve (Plant PC3) (Supplementary Tables 5, 6).

**FIGURE 7:**
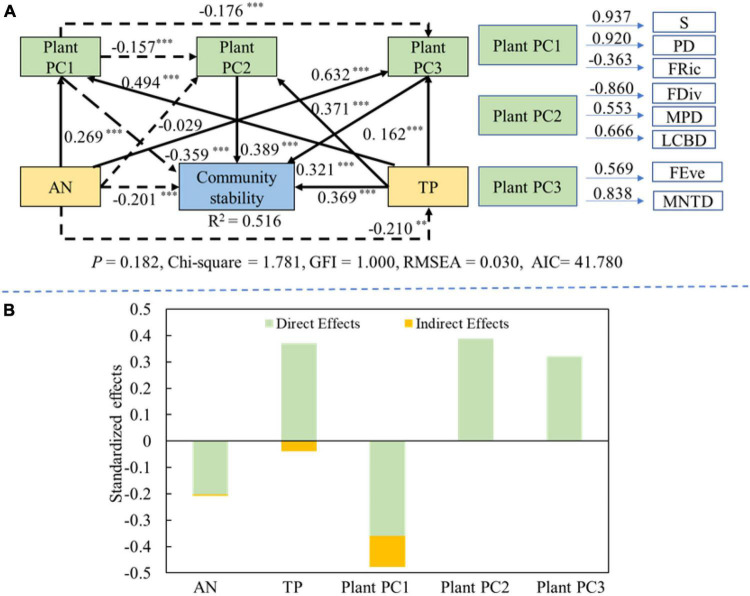
The effects of plant diversity and soil properties on the community stability. Black solid and black dashed lines indicate the positive and negative effects, respectively. Values on lines denote the standardized effect size. **(A)** Structural equation model showing the direct and indirect effects of plant diversity and soil factors on community stability. **(B)** The standardized direct and indirect effects about structural equation model. **P < 0.01; ***P < 0.001.

The SEM results showed that soil ammonium nitrogen had a significant direct negative effect on community stability (path coefficient = −0.201; *P* < 0.01). Soil total phosphorus also significant indirectly affected plant PC1 and plant PC3 to regulate community stability. Soil total phosphorus had a significant direct positive effect on the community stability (path coefficient = 0.369; *P* < 0.001), it also indirectly regulated community stability *via* affecting plant PC1, plant PC2 and plant PC3. Plant PC2 and plant PC3 had the significantly positive directly influences on the community stability (path coefficient = 0.389 and 0.321; *P* < 0.001), while plant PC1 had the significant directly negative effect (path coefficient = −0.359; *P* < 0.001) (Figure 7A). The contribution order of the influencing factors was as follows: plant PC1 (−0.477) > soil PC2 (0.389) > soil total phosphorus (0.331) > plant PC3 (0.321) > soil ammonium nitrogen (−0.208) (Figure 7B).

## Discussion

### Moderate disturbance and drought-salt stress resulted in higher stability and diversity in ecotone community than desert shrubs and riparian forest

Our results found that the stability in the ecotone community was higher than desert shrubs, and they were higher than riparian forest (Figure 2). As suggested by the “Moderate Disturbance Hypothesis,” since ecotone community was located in the transition zone between riparian forest and desert shrubs, plant diversity (species, functional diversity, and phylogenetic diversities) in this area was higher than the other two communities due to moderate disturbance (Zhao et al., 2017; Figures 3, 4). The complex community structure and relationships among plants made it more stable. Compared with riparian forest, desert shrub community mainly consisted of shrubs and herbaceous plants with stronger drought resistance. They have a short life history, abundant seeds, and high reproductive capacity, which allowed them to recover quickly after community stability is destroyed. Therefore, the stability of desert shrub community was higher than that of the riparian forest community (Chen et al., 2012). This result was consistent with the finding of the Pfisterer and Schmid on the resistance and resilience of ecosystems to drought disturbance (Pfisterer and Schmid, 2002). They found that systems with fewer species were more resistant or adaptable to environmental stresses in the arid desert regions.

Another reason for the difference in diversity among the three communities was drought and salt stress. In arid desert, the water content in the soil in the riparian forest was high due to the water replenishment from river water to the groundwater. This was beneficial to the growth for shallow-rooted herbs (Yang et al., 2017). However, on the other hand, the high groundwater level made salt more easily migrate to the soil surface with water evaporation, which leaded to the increasing degree of salt stress suffered by plants (Zheng et al., 2005). This was not conducive to the growth of shallow-rooted and salt-tolerant herbs or small shrubs (Li et al., 2019b). The trade-off between drought and salt stress limited the growth of some salt-tolerant and wet-tolerant herbaceous plants, leading to a decline in species richness in riparian forest. In terms of desert habitat, only few drought-tolerant herbs and shrubs can survive due to the water deficient, thus maintaining low biodiversity. In contrast, the ecotone community was not affected by high salinity and low soil water content (more drought stress), thus many drought-tolerant and alkali-tolerant shrubs and herbs could survive, thus leading to the highest species richness and diversity in this community (Figure 3).

Except for functional richness (FRic), the other diversity in the three communities all increased with the increase of spatial scale. This may be because larger sampling areas include more species, thus increasing the diversity. FRic reached the maximum in ecotone community at 20 m × 20 m. This may be due to functional desaturation at smaller spatial scales (10 m × 10 m), while overabundance of species may lead to functional redundancy at larger scales (50 m × 50 m). Although the number of species and phylogenetic diversity was the largest at 50 m × 50 m, the difference in response to environmental change among species may lead to a significant decline in functional richness under the influence of disturbance (Díaz and Cabido, 2001; Qiao et al., 2022; Figure 4).

### Asynchronous effect of species composition and functional traits increased community stability

An increasing number of scholars believed that higher species diversity can stabilize the ecosystem functions (Gross et al., 2014). In this study, the relationship between diversity and community stability have been studied across three spatial scales (10 m × 10 m, 20 m × 20 m, and 50 m × 50 m). The results showed that the correlation coefficient of Simpson dominance indexes to community stability were significantly larger than that of species richness (Figure 5), which indicated that the influence of dominance on community stability was greater than that of species number. This may be because the contribution of each species was uneven to community stability, and the increase of species number had not necessarily in promoting community stability (Li et al., 2022). As suggested by the “Selection Effect,” the dominant species determined ecosystem recovery process in a short time after stability disequilibrating due to their special characteristics such as the high resource utilization. An increasing of the relative abundance of dominant species will greatly increase community stability (Grman et al., 2010), because the contribution of dominant species to stability was higher than that of other species (Wayne Polley et al., 2007). Moreover, according to the “Complementary Effect,” the increase of biodiversity was beneficial to the functional complementation among species due to different specific niches among species. Niche differentiation among species can enable community to capture more resources, thus achieving stability under the premise of rational allocation of resource (Loreau and Hector, 2001). This compensatory effect can be confirmed in our results. The diversity of the ecotone community was significantly higher than those in other communities. The advantage in diversity made its community stability was higher than the other two communities.

Studies have shown that functional and phylogenetic diversities may be more closely related to community stability than species diversity (Reiss et al., 2009; Cadotte et al., 2011; Naeem et al., 2012). Our results also supported this view. There was a significant positive correlation between the MPD species and community stability in all communities (Figure 6). This suggested that high phylogenetic diversity provided more complementary functions (Ritchie et al., 2021), and higher nutrient utilization efficiency (Shang and Yan, 2017), thus improving community stability. Additionally, our results found that the RaoQ index was positively correlated with the community stability. This may indicate that the differentiation of functional niche within community can promote community stability (Mason et al., 2010). The greater difference of traits among species ensured community obtain more resources and reduce intraspecific competition, which in turn increased community stability. The functional divergence reflected the degree of niche complementarity among species in a community. Higher values of such index suggested less niche overlap among species in the community. An increase of functional divergence was conducive to maintain the community stability.

Apart from alpha diversity, beta diversity also had an non-negligible impact on community stability. It has been found that the spatial similarity of community composition (biological homogenization) may increase the spatial synchronization of ecosystem dynamics, thus reducing community stability (Olden et al., 2004; France and Duffy, 2006). Synchronization within and between communities may explain the positive biodiversity-stability relationship (Zhang et al., 2018). In this study, the changes in alpha diversity was consistent with beta diversity among three communities (ecotone community > desert shrubs > riparian forest). The consistent change between those two types of diversity was more conducive to maintaining the positive relationship between diversity and stability. This was because alpha and beta diversity can enhance the stability of regional ecosystems through “local and spatial insurance effects” (Wang and Loreau, 2016). The “local insurance effect” was originated from the asynchronous response of species to the local environment due to functional trait differentiation among species (Yachi and Loreau, 1999). The “spatial insurance effect” was caused by the asynchronous response of different communities with different species composition to the related spatial environment (Wang and Loreau, 2016). In this study, such response can be demonstrated by the performance of different species. Our results found that the responses of woody plants and herbaceous to scale changes were differed. The unsynchronized responses between these two life forms to the spatial scale were helpful to increase community stability (Figure 6). Moreover, previous studies have found that the responses of resource investment and distribution pattern to drought-salt stress differed between different plant growth types in arid desert region (Qie et al., 2018). These suggested that the asynchronous effect caused by the changes in species composition and functional traits among communities had a positive impact on the stability. The biodiversity loss and biological homogenization may damage the community stability in the arid desert ecosystem (Wang S. et al., 2021).

### Soil ammonium nitrogen and phosphorus promoted community stability by affecting plant diversity

Many studies have shown that, climatic conditions and topography are the main factors affecting plant diversity at the global scale, but the regional scale, environmental factors such as soil nutrients are considered to determine distributions of plant communities (Yu et al., 2009; Zhang et al., 2016). Such difference in scale leaded to an extremely complex relationships among environmental factors, diversity and stability, The change of community stability with spatial scale was not caused by a single environmental factor, but by the complex interaction of multiple factors (Pyšek and Lepš, 1991). Current research shown that the influence of environmental factors on the diversity-community stability relationship have the following types: positive promotion (Adams and Zhang, 2009), the effect of “single peak” curve (Zhao et al., 2007), negative restrictions (Zhang et al., 2011) and no no-significant effect. However, all of these results occurred in humid region, we did not known how relationship are existed in the arid desert region.

Our study found that soil factors not only directly affected the community stability, but also indirectly influenced it by regulating plant diversity. Moreover, the interaction among plant diversity in each dimension also regulated the variation of community stability (Figure 7). Soil ammonium nitrogen directly reduced community stability. This may be because the increased nitrogen improved the efficiency of plant use of soil nutrients, further giving some species that can use additional nitrogen an advantage in interspecific competition (Xu et al., 2015a). Soil ammonium nitrogen can positively affect the MNTD in promoting community stability, which may be because phylogenetic diversity was significantly enhanced with the increase of soil ammonium nitrogen (Sun et al., 2021). As interspecific differences in functional traits are regulated by phylogeny, the enhanced phylogenetic diversity may lead to greater niche complementarity, thus promoting community stability (Thompson et al., 2015). Many studies had found that soil phosphorus content was a key determinant of biodiversity, because it affected the structure and composition of plant communities (Lambers et al., 2011; Li et al., 2018b; Yang X. T. et al., 2022). In semi-arid and arid areas soil contained a large amount of calcium salts due to water shortage, which adsorbed and fixed phosphorus, making it difficult for plants to absorb and use phosphorus. Therefore, phosphorus was considered to be one of the major limiting factors of the ecosystem in these areas (Wang et al., 2008). In this study, soil total phosphorus had a significant positive effect on plant community stability. This may be because found the increased soil phosphorus concentration accelerated phosphorus the uptake of the phosphorus by plants (Hedin, 2004), allowing species to occupy more ecological niches, and thus increasing community stability.

As a dimension of beta diversity, LCBD represented the contribution of community composition to beta diversity. Larger LCBD showed that the community owned the high ecological specificity, and its composition greatly changed with environmental gradient or spatial scale (Dubois et al., 2020). This study found that the LCBD had a significant positive impact on community stability, suggesting that communities with high ecological uniqueness had higher stability. This may be because communities with high ecological uniqueness often had obvious differences with other communities (Dubois et al., 2020), High ecological uniqueness may utilize more resources and increase the differences of plant functional traits within the community, which in turn was more conducive to community stability. This also can be confirmed by the FDiv results. FDiv reflected the contribution of rare species to community diversity (Song et al., 2011), Higher FDiv suggested the rare species played more obviously role in community stability. In this study, FDiv had a negative and significant impact on community stability, indicating that the increase of rare species dominance would inhibit community stability. On the contrary, it confirmed the positive effect of dominant species on community stability from another aspect.

## Conclusion

Our results found community stability decreased significantly with the increase of spatial scale in the arid desert ecosystem. Community stability in the ecotone community was higher than that in desert shrubs and riparian forest. The correlation coefficient of Simpson index with community stability was significantly larger than that of species richness in all sampled communities. These suggested that the moderate disturbance and drought-salt stress resulted in higher stability and diversity in ecotone community than desert shrubs and riparian forest. The influence of species dominance on community stability was greater than the species number. Our results found that there existed obvious relationship between community stability and diversity in three sampled communities, suggested that the increase of biodiversity was beneficial to community stability due to the “Complementary Effect” of functional niches. The SEM results showed that soil ammonium nitrogen and total phosphorus not only directly affected the community stability, but also have the indirect influences by regulating the change of plant diversity. Moreover, the interaction of plant diversity in different dimension also regulated the change of community stability. Our study also found that local community with high specificity were more stable, which suggested that high specificity communities should be emphasized in diversity conservation. Although increasing plant diversity is considered an important way to maintain community stability, our results suggested that selecting suitable dominant species to enhance community stability may be a better approach.

There are also some deficiencies in this study. The functional traits only include leaf traits, but not root, hydraulic and stem traits. In arid desert, root and hydraulic traits are more advantageous than leaf traits in expressing plant adaptability to environment, especially to drought stress. In addition, only interspecific differences were used to analyze the effect of trait differentiation on community stability in this study, while intraspecific variation of traits was ignored. As many studies have confirmed that the intraspecific variation of traits also plays an important role in ecosystem functioning (Arshad and Yan, 2018; Bello et al., 2021; Jiang M. et al., 2022). Moreover, this study only analyzed the changes of community stability with spatial scale and its influencing factors. It is well known that functional traits, niche and species turnover also vary across temporal scales (Furness et al., 2021; Xu et al., 2021). For example, ephemeral plants are common in desert communities in early spring. The higher abundance of these plants leads to higher plant diversity of community at that time than at other times of year. But after spring, their mortality greatly reduces the diversity and stability of the community. Therefore, the temporal and spatial changes of the relationship between community stability and plant diversity should be synchronous analyzed in future studies. It has been found in recent years that the soil microorganisms have a particularly important effect on plant community composition, thus may also play an important role in maintaining community stability (Zhang et al., 2021). Therefore, the influence of soil microbe-plant interaction on community stability also need to be considered in future studies.

## Data availability statement

The original contributions presented in this study are included in the article/Supplementary material, further inquiries can be directed to the corresponding authors.

## Author contributions

L-MJ, KS, DH, JZ, and G-HL conceived and designed the experiments. L-MJ, KS, DH, and JZ performed the experiments. L-MJ analyzed the data and wrote the manuscript. X-DY improved the quality of the article. All authors contributed to the article and approved the submitted version.
