# Supplementary material for: Different contributions of plant diversity and soil properties to the community stability in the arid desert ecosystem
Source: Front Plant Sci. 2022 Aug 25;13:969852. doi: 10.3389/fpls.2022.969852 (PMC9453452; doi:10.3389/fpls.2022.969852)
Supplement: Supplementary file 1 [file Data_Sheet_1.docx]

Different contributions of plant diversity and soil properties to community stability in the arid desert ecosystem

**Table S1 Species composition of the riparian forest community**

| Species name | Genus | Family |
| --- | --- | --- |
| *Haloxylon ammodendron* | Haloxylon | Chenopodiaceae |
| *Populus euphratica* | Populus | Salicaceae |
| *Halimodendron halodendron* | Halimodendron | Leguminosae |
| *Nitraria roborowskii* | Nitraria | Zygophyllaceae |
| *Reaumuria soongorica* | Reaumuria | Tamaricaceae |
| *Lycium ruthenicum* | Lycium | Solaneae |
| *Apocynum venetum* | Apocynum | Apocynaceae |
| *Phragmites australis* | Phragmites | Gramineae |
| *Sonchus oleraceus* | Sonchus | Compositae |
| *Suaeda microphylla* | Suaeda | Chenopodiaceae |
| *Psathyrostachys juncea* | Psathyrostachys | Gramineae |
| *Alhagi sparsifolia* | Alhagi | Leguminosae |
| *Salsola collina* | Salsola | Chenopodiaceae |
| *Glycyrrhiza uralensis* | Glycyrrhiza | Leguminosae |

**Table S2 Species composition of ecotone community**

| Species name | Genus | Family |
| --- | --- | --- |
| *Nitraria roborowskii* | Nitraria | Zygophyllaceae |
| *Karelinia caspia* | Karelinia | Compositae |
| *Tamarix chinensis* | Tamarix | Tamaricaceae |
| *Populus euphratica* | Populus | Salicaceae |
| *Aeluropus pungens* | Aeluropus | Gramineae |
| *Halocnemum strobilaceum* | Halocnemum | Chenopodiaceae |
| *Kalidium foliatum* | Kalidium | Chenopodiaceae |
| *Haloxylon ammodendron* | Haloxylon | Chenopodiaceae |
| *Seriphidium terrae-albae* | Seriphidium | Compositae |
| *Atriplex centralasiatica* | Atriplex | Chenopodiaceae |
| *Salsola collina* | Salsola | Chenopodiaceae |
| *Alhagi sparsifolia* | Alhagi | Leguminosae |
| *Suaeda glauca* | Suaeda | Chenopodiaceae |
| *Suaeda microphylla* | Suaeda | Chenopodiaceae |
| *Apocynum venetum* | Apocynum | Apocynaceae |
| *Reaumuria soongarica* | Reaumuria | Tamaricaceae |
| *Phragmites australis* | Phragmites | Gramineae |

**Table S3 Species composition of desert shrubs community**

| Plant Species name | Genus | Family |
| --- | --- | --- |
| *Halocnemum strobilaceum* | Halocnemum | Chenopodiaceae |
| *Reaumuria soongarica* | Reaumuria | Tamaricaceae |
| *Nitraria roborowskii* | Nitraria | Zygophyllaceae |
| *Alhagi sparsifolia* | Alhagi | Leguminosae |
| *Calligonum mongolicum* | Calligonum | Polygonaceae |
| *Seriphidium terrae-albae* | Seriphidium | Compositae |
| *Horaninowia ulicina* | Horaninowia | Chenopodiaceae |
| *Phragmites australis* | Phragmites | Gramineae |
| *Salsola collina* | Salsola | Chenopodiaceae |
| *Suaeda glauca* | Suaeda | Chenopodiaceae |

**Table S4 The collinearity analysis results obtained by multiple regression analysis**

| types | factors | Collinearity statistics | | |
| --- | --- | --- | --- | --- |
|  |  | tolerance | VIF | |
| plant diversity | Species richness | 0.126 | | 7.934 |
|  | Simpson | 0.059 | | 17.021 |
|  | Shannon Wiener | 0.023 | | 44.309 |
|  | functional richness | 0.836 | | 1.196 |
|  | functional evenness | 0.817 | | 1.224 |
|  | functional divergence | 0.594 | | 1.683 |
|  | functional dispersion | 0.018 | | 56.303 |
|  | RaoQ | 0.025 | | 40.661 |
|  | phylogenetic richness | 0.118 | | 8.449 |
|  | mean pairwise phylogenetic distance | 0.127 | | 7.879 |
|  | mean nearest taxa distance | 0.270 | | 3.708 |
|  | Local Contribution to Beta Diversity | 0.766 | | 1.306 |
| soil factors | pH | 0.076 | | 13.110 |
|  | soil water content | 0.045 | | 22.417 |
|  | soil salinity content | 0.058 | | 17.355 |
|  | soil organic carbon | 0.044 | | 22.818 |
|  | soil total phosphorus | 0.203 | | 4.930 |
|  | soil available phosphorus | 0.040 | | 24.734 |
|  | soil total nitrogen | 0.039 | | 25.379 |
|  | soil [ammonium nitrogen](D:/Drivers/Dict/9.0.1.1/resultui/html/index.html#/javascript:;) | 0.196 | | 5.089 |
|  | soil [nitrate nitrogen](D:/Drivers/Dict/9.0.1.1/resultui/html/index.html#/javascript:;) | 0.046 | | 21.592 |

**Table S5 Percentage of total variance of principal component analysis**

| component | eigenvalue | Variance percentage | Cumulative variance percentage |
| --- | --- | --- | --- |
| 1 | 2.273 | 28.411 | 28.411 |
| 2 | 1.581 | 19.764 | 48.175 |
| 3 | 1.232 | 15.403 | 63.578 |

**Table S6 The Component matrix**

|  | Principal component | | |
| --- | --- | --- | --- |
| factors | PC1 | PC2 | PC3 |
| S | 0.937 | -0.038 | -0.233 |
| FRic | -0.363 | 0.259 | -0.127 |
| FEve | 0.131 | 0.125 | 0.569 |
| FDiv | 0.059 | -0.860 | -0.052 |
| PD | 0.920 | 0.043 | 0.201 |
| MPD | 0.538 | 0.553 | 0.376 |
| MNTD | -0.080 | -0.218 | 0.838 |
| LCBD | -0.047 | 0.666 | -0.084 |


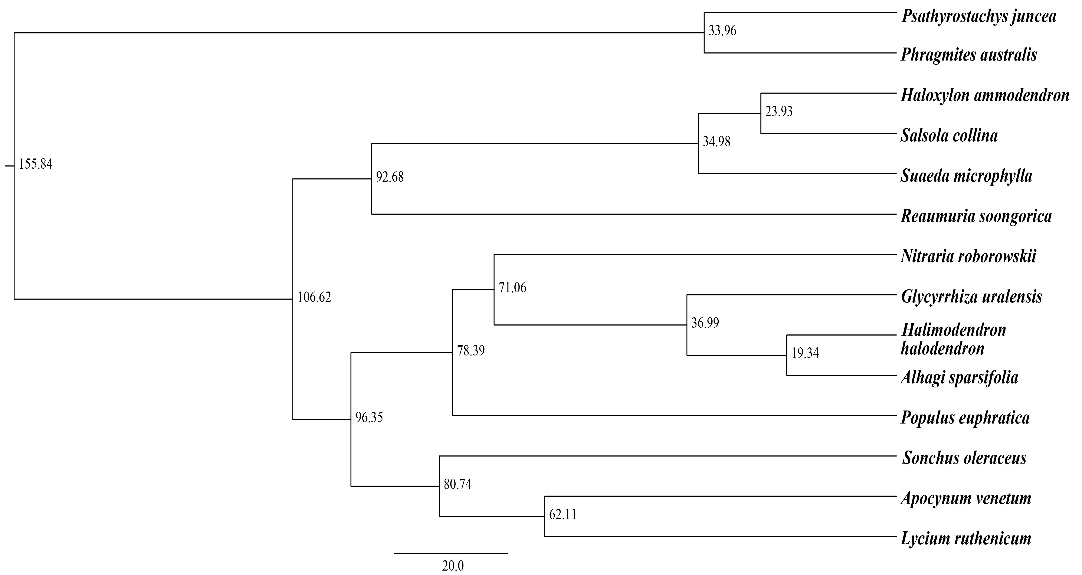


**Figure S1 The phylogenetic tree of riparian forest community**


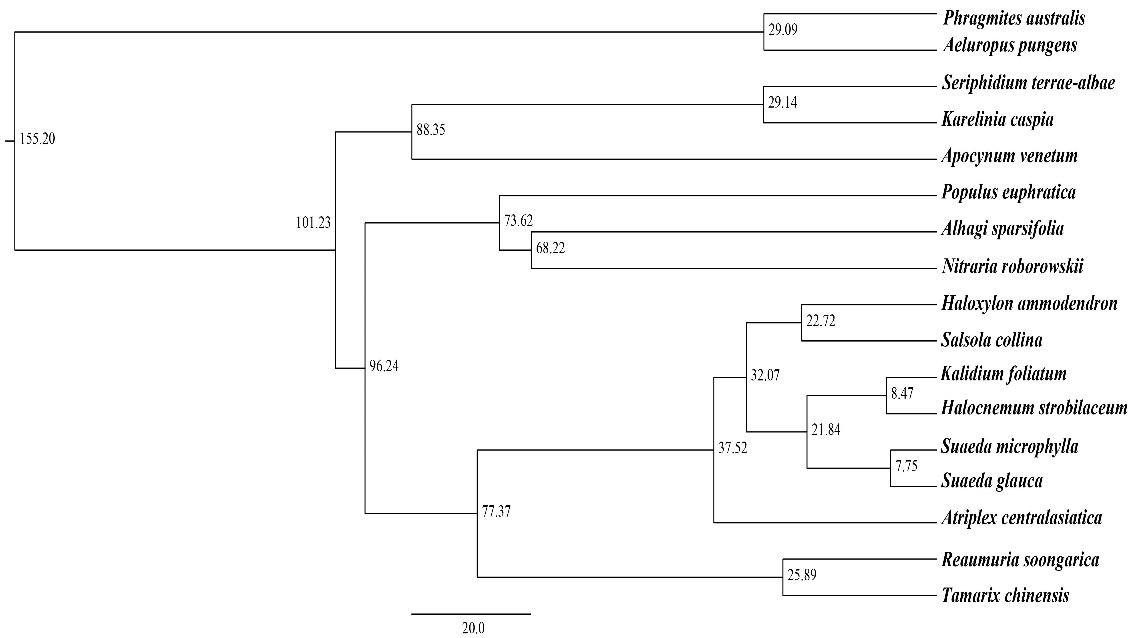


**Figure S2 The phylogenetic tree of ecotone community**


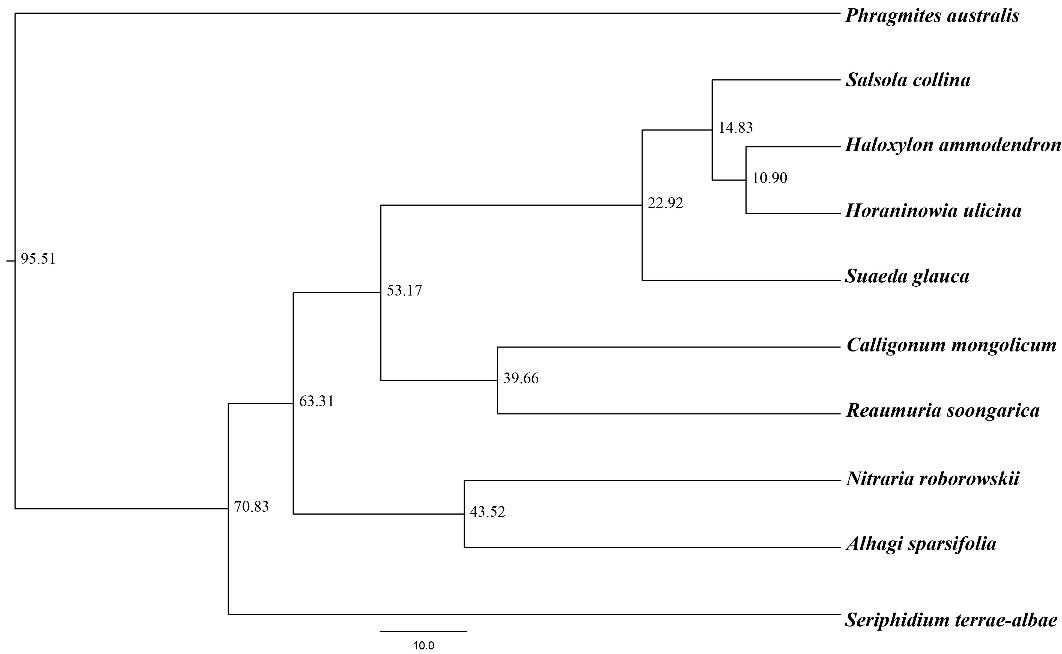


**Figure S3 The phylogenetic tree of desert shrubs community**


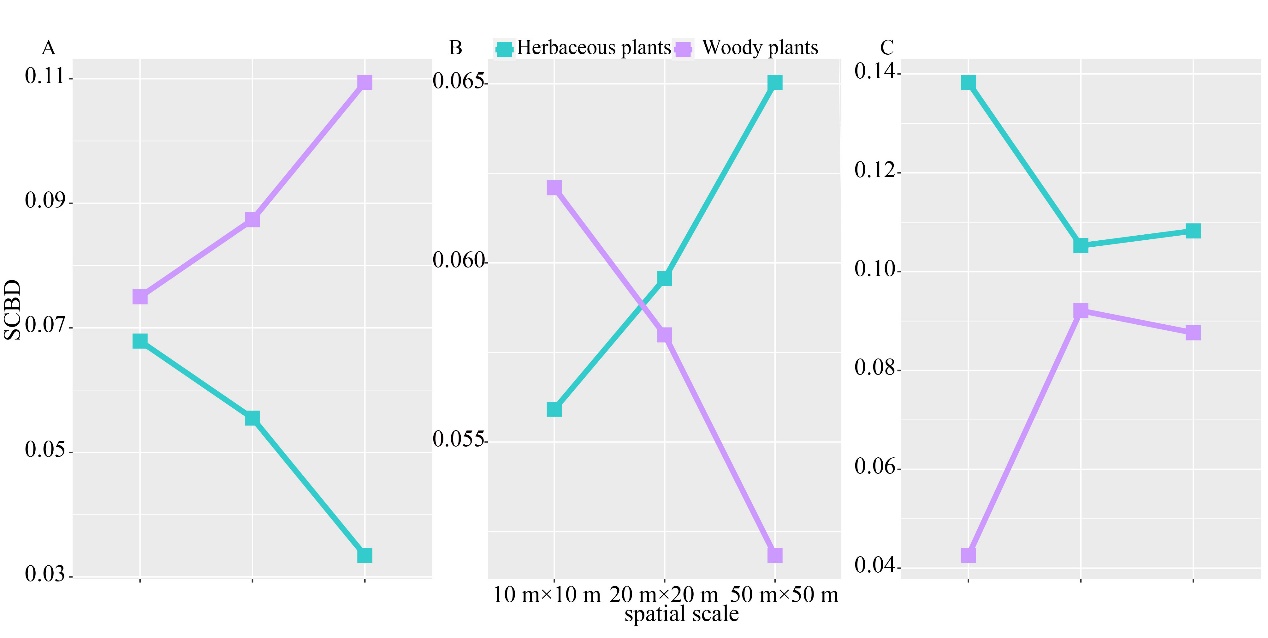


**Figure S4. Difference in the uniqueness change across** [**spatial**](D:/Drivers/Dict/9.0.1.1/resultui/html/index.html#/javascript:;) **scales between herbaceous and woody plants. A:** [**riparian forest**](C:/Users/ABC/AppData/Local/Youdao/Dict/Application/9.0.4.0/resultui/html/index.html#/javascript:;)**; B: ecotone community; and C: desert shrubs.**
